# Prospective study on outcomes of endotherapy for pancreatic divisum in patients of recurrent acute pancreatitis

**DOI:** 10.1055/a-2641-5532

**Published:** 2025-07-24

**Authors:** Amol Vadgaonkar, Nagesh Kamat, Ankit Dalal, Gaurav Patil, Sanil Parekh, Sehajad Vora, Amit Maydeo

**Affiliations:** 1 81727 Institute of Gastrosciences, Sir HN Reliance Foundation Hospital and Research Centre, Mumbai, Maharashtra, India

**Keywords:** Pancreatobiliary (ERCP/PTCD), endotherapy, recurrent acute pancreatitis, pancreatic divisum

## Abstract

**Background and study aims:**

Minor papilla endotherapy success rate is highly variable for pancreatic divisum (PD) among recurrent acute pancreatitis (RAP) patients due to frequent relapses. Therefore, we assessed effectiveness and predictors of successful endotherapy.

**Patients and methods:**

This was a prospective observational study of patients with RAP who underwent minor papilla sphincterotomy and prophylactic stenting for PD. Technical success was minor papilla cannulation and successful procedure completion. Primary and secondary outcomes were improvement in recurrent episodes of pain with reduction in visual analogue scale (VAS) score > 50% from baseline and occurrence of chronic pancreatitis (CP) at 12 months, respectively. Predictors of success were assessed by logistic regression.

**Results:**

Ninety-four patients, with median age (interquartile range) 29.5 years (23.7–40.2); the majority male (62 [65.9%]), successfully underwent endotherapy. Typical clinical presentation was abdominal pain in 87 patients (92.5%). The primary outcome was achieved in 65 patients (69.1%). The average number of endoscopic retrograde cholangiopancreatography (ERCP) sessions was two; technical success was achieved in 88 patients (93.6%). Post-ERCP pancreatitis was the most common adverse event (AE) in 10 patients (10.6%). Signs of CP were seen in 11 patients (11.7%) and mean follow-up period was 12.8 ± 1.3 months. Presence of smoking (adjusted odds ratio [AOR] 0.027, *P* = 0.001) and recurrent attacks of RAP after index ERCP (AOR 0.169, *P* < 0.001) had lower odds of successful endotherapy outcomes.

**Conclusions:**

Minor papilla endotherapy for RAP significantly improved VAS scores at 12 months among 69.1% of patients with acceptable AEs. Early CP was seen in 11.7% of patients. (Unique identifier: CTRI/2019/05/019332).

## Introduction

Pancreatic divisum (PD) is a congenital pancreatic anomaly seen in about 10% of patients 1 2 . Patients with PD are often asymptomatic, but some are at substantial risk of recurrent abdominal pain 3 . These patients have recurrent bouts of seemingly recurrent acute pancreatitis (RAP) 4 5 . Due to the small ampullary orifice, significant intrapancreatic dorsal ductal pressure can arise during active pancreatic secretion 6 7 . This can lead to inadequate drainage, pancreatitis, ductal distension, discomfort, and pain 7 . Patients who have PD with RAP usually have associated genetic mutations 8 . Identifying the cause helps to direct therapy. Minor papilla endoscopic sphincterotomy improves pain in PD 9 10 , but the true benefit and its therapeutic significance remain controversial and need clarity. These patients need to be identified and treated early before they develop progressive signs of malabsorption, diabetes, and chronic pancreatitis, 11 the treatment of which is costly 12 . The eventual therapy is pancreatic surgery 13 14 . Endoscopic and surgical modalities for PD have not been compared. The majority of the evidence is derived from small, uncontrolled, retrospective studies. Therefore, this study aimed to identify the benefit of pancreatic sphincterotomy and prophylactic stenting for PD among patients with RAP in this prospective study.

## Patients and methods

### Study population

Consecutive patients with RAP who presented during the study tenure from June 2019 to May 2024 were reviewed and assessed for eligibility in this prospective observational study. The last patient follow-up was in May 2024, and the first and last patients were enrolled in June 2019 and June 2023, respectively. This research was carried out by Good Clinical Practice and the 1975 Helsinki Declaration on Human Rights, as updated in 2013 15 . The Institutional Ethics Committee evaluated and granted ethical approval for the study protocol and all procedures in this study (IEC/OA-26/18). Prior to enrollment, each patient provided written informed consent. Before the first participant was recruited, the trial was registered in the Clinical Trials Registry of India, a publicly accessible database. - URL: http://ctri.nic.in (Unique identifier: CTRI/2019/05/019332). Every author had access to the study data and gave their final article approval.

### Eligibility

Inclusion criteria were patients of RAP with PD (diagnosed on magnetic retrograde cholangiopancreatography and endoscopic ultrasound) aged 18 to 60 years. Exclusion criteria were patients with prior minor papilla sphincterotomy/pancreatic surgery, chronic pancreatitis, main pancreatic duct stricture, other structural etiology of pancreatitis, plan of long-term travel during the study period, pancreatic malignancy, pregnancy, and refusal of consent.

### Preprocedure requirements and symptom evaluation

All patients were evaluated for etiology of pancreatitis. A routine clinical history was conducted for patient symptomatology, medications, hospitalizations, and social history. All patients had to undergo routine laboratory investigations and electrocardiogram for anesthesia fitness and radio imaging (not repeated if done within the past 2 weeks). The pancreatic duct was considered dilated if the diameter was greater than 3 mm in the head, 2 mm in the body, and 1 mm in the tail of the pancreas. Serum investigations included complete blood count, liver function tests, lipid profile, serum amylase, lipase, calcium, creatinine, immunoglobulin G4 (IgG4), and parathyroid hormone. Patients had to undergo endoscopic ultrasound (EUS) to look for microlithiasis and liver function tests to check the requirement for a biliary endoscopic retrograde cholangiopancreatography (ERCP). A visual analog scale (VAS) was used for pain scores before endotherapy. By placing a handwritten mark on a 10-cm line, patients were asked to rate their level of abdominal pain, with no pain on the left end of the scale and the worst pain on the right 16 . The maximum timeframe between laboratory investigations while considering the patient for study inclusion was 1 week.

### Endoscopic retrograde cholangiopancreatography

**Fig. 1:**
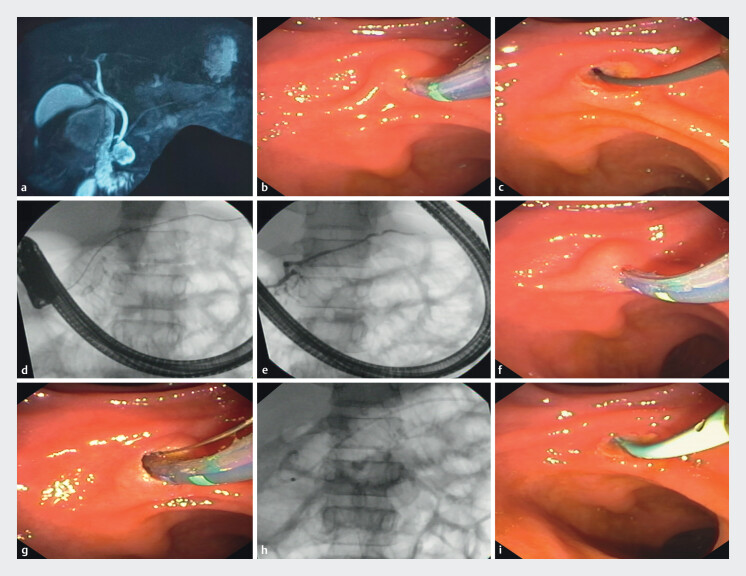
**a** MRCP showing complete pancreatic divisum. **b** Endoscopy showing minor papilla cannulation. **c** Hydrophilic guidewire (Terumo) insertion in the pancreatic duct. **d** Fluoroscopy showing guidewire in the pancreatic duct. **e** Pancreatogram after injecting radiopaque contrast. **f, g** Minor papilla sphincterotomy. **h** Fluoroscopic image of pancreatic duct stenting. **i** Endoscopic image of prophylactic pancreatic duct stenting.

Procedures were performed under total intravenous (IV) anesthesia with patients in the supine position. All patients received diclofenac suppository (Jonac 100 mg) per rectally 30 min before the onset of ERCP. Patients underwent ERCP according to standardized treatment protocol with a duodenoscope [TJF-Q190V Olympus, Japan) under fluoroscopic guidance. The minor papilla was cannulated using a triple-lumen sphincterotome (Clevercut 3V [Olympus]). A pancreatogram was taken after injecting radiopaque contrast-diluted diatrizoate meglumine (Trazograf 76%) to look at pancreatic duct characteristics and relieve obstruction at the level of the minor papilla. A 0.032-inch J-angled tip hydrophilic guidewire (Terumo) was advanced in the pancreatic duct ( Fig. 1 **a-i** ). Minor papilla sphincterotomy was done over the guidewire as per the discretion of the treating endoscopist. A prophylactic 5F single pigtail pancreatic stent (7/10/12 cm, Cook Medical, United States) was placed in the pancreatic duct in all patients. Stent length was selected based on distance from the papilla to the tail/body of the pancreas plus an additional 1 cm (measured by passing the sphincterotome to the tail/body and slowly withdrawing it till the papilla) under fluoroscopy.

All patients were admitted and kept nil per os. On day 1, patients were given parenteral proton pump inhibitors (pantoprazole), IV fluids, metoclopramide, and analgesics as appropriate (paracetamol and diclofenac suppository for mild to moderate pain, pentazocine for severe pain). On day 2, clear liquids were started as tolerated and were asked to switch to a soft semisolid diet over the next 3 to 4 days before moving to solid food. Patients were ideally discharged 48 to 72 hoiurs after the procedure. Patients were requested to keep a medication diary listing all the ingested drugs. In the event of discomfort, fever, vomiting, or symptom recurrence, patients were instructed to contact the study site.

### Follow up

Unless the patient showed fresh symptoms, the planned follow-up was conducted at 1, 6, and 12 months. A phone call was made to all patients to remind them of the planned follow-up (± 3 days). At 1 month, patients were assessed clinically. If they had improvement in pain, the pancreatic stents were checked for spontaneous passage else removed (stent-free trial) at ERCP, followed by a pancreatogram to look for pancreatic duct patency. If the stent had spontaneously dislodged (assessed on fluoroscopy), there was no need for another ERCP in the case of asymptomatic patients. Patients were interviewed for symptoms, and a VAS was filled (12 months). The time duration a patient was involved in the study was 1 year following study inclusion.

If the patient had pain, the severity was assessed. Ultrasound imaging, serum amylase, and lipase were used to check for pancreatitis, and appropriate analgesics were given. Once pain subsided, patients underwent ERCP to look for minor papilla stenosis, sphincterotomy site bleeding, stent migration, and possible restenting. Patients were then followed thereafter according to clinical practice.

### Outcomes

The primary outcome included improvement in the intensity of pain as reported by the patient on VAS score with a reduction > 50% (responders) from baseline at 1 year. The secondary outcome was occurrence of chronic pancreatitis 1 year after endotherapy.

### Definitions

RAP was defined as ≥ 2 unexplained episodes of acute pancreatitis without evidence of chronic pancreatitis (CP) on imaging 17 .

Acute pancreatitis was defined as either radiographic evidence consistent with acute pancreatitis or a fresh onset of abdominal pain consistent with pancreatic type with rise in serum lipase or amylase > three times the upper limit of normal 18 .

Chronic pancreatitis was defined as characteristic CP changes on imaging (MRCP) and EUS 19 . Technical success was selective minor papilla cannulation, sphincterotomy, and prophylactic dorsal duct stenting at index ERCP.

### Safety

AEs were recorded as per the American Society for Gastrointestinal Endoscopy lexicon 20 . AEs were considered as mild (mild abdominal pain, nausea, vomiting), moderate (stent migration, stent occlusion, post-ERCP pancreatitis), or severe (events requiring Intensive Care Unit stay/urgent surgery, bleeding requiring blood transfusion, sepsis, or death)

### Changes to methods after trial commencement

Patients had to screen negative for COVID-19 pneumonia for inclusion in the trial. The 2-year planned study period (including follow-up) was extended for 3 years.

### Sample size calculation and statistical analysis

Prevalence of PD was 10% in pancreatitis patients 21 22 . The sample size was calculated 23 by Cochran's formula using this value and assuming the following parameters: confidence level at 95%, precision level of ± 10%, and level of significance of 0.05; the sample size was found to be 35. After correcting 12% for missing data and dropout, the minimum representative sample size was 40. IBM Statistical Package for the Social Sciences (SPSS) [version 26.0, Professional] for Windows was used to conduct the statistical analysis (IBM Corp., Armonk, New York, United States). Descriptive statistics are applied to continuous variables. Frequency and percentage are reported for categorical variables. When comparing quantitative data recorded between binomial qualitative variables, the Mann-Whitney U test was used if the data failed the "Normality" test, and the Unpaired t-test was used if the data passed the "Shapiro-Wilk Normality test." The Chi-square test was applied for nonparametric data, and paired t-test for follow-up VAS scores comparison. Bivariate analysis was conducted to identify predicting factors for successful endotherapy and significant factors were further analyzed by logistic regression analysis. Variables that were correlated (multicollinearity) to the target variable or to each other were excluded from the final regression analysis. Results are graphically represented as box plots. *P* <.05 was considered statistically significant.

## Results

### Patient demographic characteristics

Ninety-four consecutive patients, with a median (interquartile range [IQR]) age of 29.5 years (23.7–40.2); the majority male, 62 (65.9%), successfully underwent endotherapy for PD in RAP ( Table 1 ). We did not encounter any cases of Santorinicele. The common clinical presentation was abdominal pain in 87(92.5%) patients. Patients had a median (IQR) previous episode of RAP of 3(2–3). The median (IQR) duration of RAP was 3 years (3–4). Most patients, 63 (67%), had a non-dilated duct on MRCP and complete PD in 65 patients (69.1%). Mean postoperative stay was 3 ± 0.8 days. Mean follow-up period was 12.8 ± 1.3 months ( Table 2 ). No patients had undergone biliary ERCP or cholecystectomy prior to study inclusion.

**Table:** **Table 1** Baseline patient characteristics.

Variable	N=94
Age, median (IQR), y	29.5 (23.7–40.2)
Gender, male, n (%)	62 (65.9)
Male:female	1.9:1
Clinical presentation*, n (%)	
Abdominal Pain	87 (92.5)
Nausea	56 (59.6)
Vomiting	48 (51.1)
Previous episodes of RAP, median (IQR)	3 (2–3)
Duration of the disease (y), median (IQR)	3 (3–4)
Social habits, n (%)	
Smoking	10 (10.6)
Type of pancreatic duct, n (%)	
Dilated duct	31 (33)
Non dilated duct	63 (67)
Laboratory parameters (mean ± SD)	
Hemoglobin	12.5 ± 1.4
Leukocytes	6932.5 ± 2363
Platelets	296480.2 ± 114035.1
Total bilirubin	1.04 ± 0.3
Aspartate transaminase	38.4 ± 20.5
Alanine transaminase	41.1 ± 31.5
Alkaline phosphatase	132.5 ± 30.6
Serum creatinine	0.9 ± 0.2
Serum amylase	407.5 ± 209.2
Serum lipase	467.6 ± 330.4
Serum calcium	9 ± 0.7
Total cholesterol	131 ± 25
Low-density lipoproteins	111.7 ± 21.4
Triglycerides	131.5 ± 50.5
Glycosylated hemoglobin	5.6 ± 0.3
Parathyroid hormone	87.4 ± 27.8
Immunoglobulin G4	31 ± 10.6
Anatomic variations, n (%)	
Complete pancreatic divisum	65 (69.1)
Incomplete pancreatic divisum	29 (30.9)
* Some patients may have more than one finding. IQR, interquartile range; RAP, recurrent acute pancreatitis; SD, standard deviation.	

**Table:** **Table 2** Characteristics of patients who underwent endotherapy for pancreatic divisum.

Variable	N = 94
VAS Score before ERCP, mean ± SD	7.6 ± 0.8
VAS Score at 12 months, mean ± SD	3.3 ± 1.4
Technical success, n (%)	88 (93.6)
Adverse events, n (%)	
Post ERCP pancreatitis	10 (10.6)
Sphincterotomy site bleed	4 (4.2)
Migrated stent	1 (1.1)
Primary outcome: > 50% reduction in VAS score, n (%)	65 (69.1)
Postoperative stay, mean ± SD, days	3 ± 0.8
ERCP sessions, n (%)	
2 ERCPs	64 (68.1)
3 ERCPs	27 (28.7)
≥4 ERCPs	3 (3.2)
Recurrent episodes after index ERCP, n (%)	
One episode	15 (15.9)
Two episodes	19 (20.2)
Three episodes	10 (10.6)
Four episodes	3 (3.2)
MRI/EUS signs of CP, n (%)	11 (11.7)
Follow-up, mean ± SD, months	12.8 ± 1.3
CP, chronic pancreatitis; ERCP, endoscopic retrograde cholangiopancreatography; EUS, endoscopic ultrasound; MRI, magnetic resonance imaging; SD, standard deviation.	

### Outcomes of endotherapy

#### Primary outcome

**Fig. 2:**
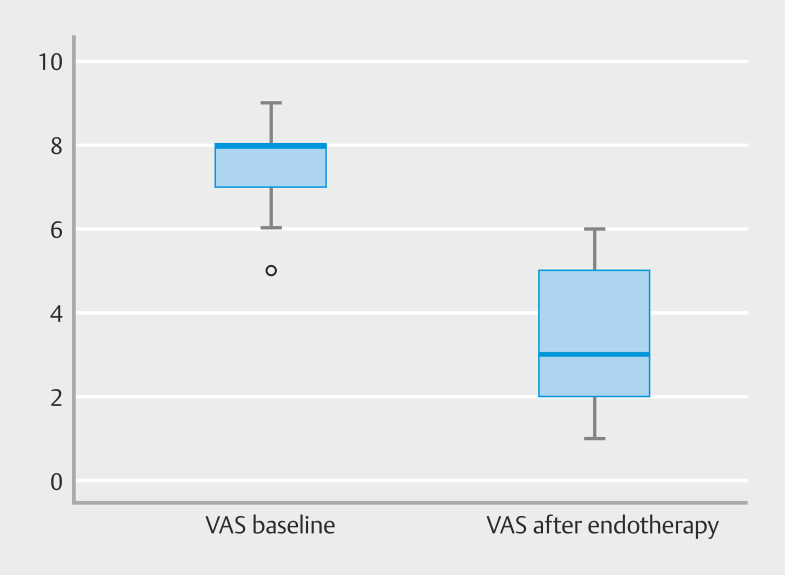
Box plot showing VAS score at baseline and after endotherapy.

Reduction of >50% of VAS score from baseline was seen in 65 patients (69.1%) ( Table 3 ). Mean VAS score among these patients was significantly lower than those who did not have > 50% reduction in VAS score (2.5 ± 0.7 vs 5.1 ± 0.6, respectively; *P* = 0.001) ( Fig. 2 ). The average number of ERCP sessions was two; technical success was achieved in 88 patients (93.6%). Six patients (6.4%) needed a repeat session after 48 to 72 hours due to failed cannulation in the presence of minor papillary edema/ inability to identify the papillary opening. The procedures were successful among these patients at the second attempt. At follow-up, due to repeated episodes of RAP (minor papilla re-stenosis, retro-ampullary narrowing), 30 patients (31.9%) needed at least three or more ERCP sessions.

**Table:** **Table 3** Bivariate analysis for primary outcome.

Variable	Pain relief N = 65	Pain persists N = 29	*P* value
Age, mean ± SD	34.1 ± 11.5	26.7 ± 7.6	**0.002**
Gender, n (%)			
Males	40 (61.5)	22 (75.9)	0.176
Females	25 (38.5)	7 (24.1)	
Smoking, n (%)			
Yes	2 (3.1)	8 (27.6)	**< 0.001**
No	63 (96.9)	21 (72.4)	
Previous episode of RAP, mean ± SD	2.8 ± 0.8	2.7 ± 0.6	0.659
Type of pancreatic duct, n (%)			
Dilated duct	23 (35.4)	8 (27.6)	0.458
Nondilated duct	42 (64.6)	21 (72.4)	
Anatomic variation, n (%)			
Complete divisum	57 (87.7)	8 (27.6)	**< 0.001**
Incomplete divisum	8 (12.3)	21 (72.4)	
Recurrent episodes after index ERCP, mean ± SD	0.4 ± 0.8	2.3 ± 0.9	**< 0.001**
ERCP, endoscopic retrograde cholangiopancreatography; RAP, recurrent acute pancreatitis; SD, standard deviation.			

In patients with better pain relief (responders), mean age was significantly higher, mean recurrent episodes after the index ERCP were significantly lower, and a significantly higher proportion of patients had complete divisum ( Table 3 ). There was a significant difference in the number of patients who smoked (3.1% vs 27.6%, *P* < 0.001) respectively ( Table 3 ) and the number of patients (3.1% vs 31%, *P* < 0.001) who developed CP, respectively. There was no significant difference among gender, previous mean episodes of RAP and the presence of a dilated pancreatic duct.

#### Secondary outcome

EUS signs of early CP were seen among 11 patients (11.7%). When risk of developing CP was studied – these patients had a lower mean age (25.4 ± 9.6 vs. 32.7 ± 10.9, *P* = 0.040), a higher proportion of smokers (72.7% vs 2.4%, *P* < 0.001) and a higher proportion of patients with a dilated pancreatic duct (63.6% vs 28.9%, *P* = 0.021). There was no significant difference among gender, previous mean episodes of RAP, and anatomic variation in divisum. CP occurred more frequently in patients with complete PD, but it was insignificant; *P* = 0.674.

### Predictors of successful endotherapy

Presence of smoking (adjusted odds ratio [AOR] 0.027, 95% confidence interval [CI] 0.012–0.771, *P* = 0.001) and recurrent attacks of RAP after index ERCP (AOR 0.169, 95% CI 0.079–0.361, *P* < 0.001) had lower odds of successful endotherapy outcome ( Table 4 ).

**Table:** **Table 4** Binary logistic regression model to identify predictors of successful endotherapy for pancreatic divisum.

Variable	AOR	*P* value	95 % CI	
			Lower	Upper
Age	1.037	0.369	0.958	1.122
Smoking	0.096	**0.027**	0.012	0.771
Recurrent attacks of RAP after index ERCP	0.169	**< 0.001**	0.079	0.361
Constant	2.347	0.098		
AOR, adjusted odds ratio; CI, confidence interval; ERCP, endoscopic retrograde cholangiopancreatography; RAP, recurrent acute pancreatitis.				

### Adverse events

Post-ERCP pancreatitis (mild) was the most common AE in 10 patients (10.6%) ( Table 2 ). They were managed with IV fluids and analgesics. Sphincterotomy site bleed was seen in four patients (4.2%), which was immediately identified and treated with balloon tamponade (n = 3, mild), whereas one patient (moderate) needed an injection of diluted adrenaline (1:10000 injected 0.5–1 mL) at the bleeding site. Patients continued to be monitored. One patient (1.1%) (mild) had stent migration which was removed, and restenting was performed.

## Discussion

By prospectively following up on patients with RAP, we studied usefulness of minor papilla endotherapy and stenting among symptomatic patients of PD. Technical success in our study was achieved in 88 patients (93.6%) 24 . This could be mainly due to excluding CP patients with stricturing disease, contributing to good technical success. The decent technical success is also partly attributed to a well sedated patient with the help of optimal analgesia obtained through limited use of nalbuphine/buprenorphine. It is preferable to diagnose PD before attempting cannulation to avoid prolonged major papilla attempts, because cannulation may still be difficult in some circumstances. The pooled technical success rate of endotherapy in individuals with PD was 92% in a recent meta-analysis of 27 trials involving 1355 patients 25 . In the present study, endotherapy significantly reduced recurring pain episodes in 65(69.1%) patients at 1 year. These results are similar to previous studies, reporting success rates of about 70%, using outcome measures such as reduction in pain episodes 26 27 . In a meta-analysis, endotherapy was successful for PD with a pooled efficacy rate of 67.5% 28 . In another meta-analysis, the pooled clinical success rate was only 65% 25 . Numerous indicators of clinical success have been employed in past studies, such as decreased pain scores, complete or partial pain relief, narcotic analgesics, emergency room visits, and duration of hospitalization. Assessment of clinical success is made more difficult by the varied definitions. Despite good technical success with endotherapy in PD, clinical success seems to be limited over time. A common cause of recurring episodes of AP in many patients is pancreatic orifice stenosis, which necessitates further treatment. The pathway through the major papilla is tightly angulated, and the accessory pancreatic duct forms a loop, which makes therapy difficult. The minor papilla is straighter and facilitates easier stent placement.

EUS signs of CP 29 were seen during follow-up in 11 patients (11.7%). In the group that experienced good pain reduction, there were notably fewer EUS signs of CP (3.1%) among all patients who received endotherapy. Changes associated with CP appeared in these patients after they had the disease for a considerable amount of time, given that median duration of symptoms at baseline was about 3 years. Ductal drainage brought about by endotherapy may have reduced or at least slowed the progression of CP, supporting the theory that outflow obstruction may have contributed to development of CP. These findings support use of endotherapy in PD patients who have experienced episodes of pancreatitis in the past. Our study showed no signs of pancreatic ductal stricturing events or pancreatic stones.

We identified potential predictors of successful endotherapy. Two variables were noted to be associated with outcomes: recurrent attacks of RAP after index ERCP and absence of smoking. The findings should be interpreted cautiously due to the small number of evaluated patients. Continuous smoking is a known risk factor predicting relapse after AP 30 . Patients with younger age tended to have poorer response rates, although the difference was insignificant. Borak et al. showed that younger age (median 46.5 vs 58 years) and CP independently predicted a lower chance of success 26 . Studies have shown that failure of clinical success after technically successful ERCP was male sex 31 . However, we did not find any gender association with outcomes. Recurrent attacks of AP after index ERCP for PD are also known to progress the disease towards CP 32 .

PD occurs in 2.7% to 22.0% of patients 33 . Although most patients remain asymptomatic, a small proportion become symptomatic with RAP, contributing to chronic abdominal pain with or without radiographic evidence of pancreatitis. The underlying mechanism is the combination of PD and minor papillary stenosis impeding the outflow of pancreatic secretion (ductal obstruction) into the duodenum, resulting in obstructive pancreatitis and chronic pain 34 . It is likely that stenting may decrease risk of post-ERCP pancreatitis and allows for adequate drainage in some patients. The proportion of post-ERCP pancreatitis (11.3%) is consistent with a reported rate of 8% to 20% in most endotherapy patients 35 . The slightly lower incidence could be attributed to limited use of contrast medium to minimize the filling pressure, which may have decreased risk of post-ERCP pancreatitis.

The main strength of this study is long-term follow-up with a decent sample size. Symptomatic PD is not frequent, and high-quality evidence is lacking. The limitation is that the data available come from a single center. Genetic factors predispose patients with pancreatic disease to intermittent obstruction. We did not identify genetic mutations in these patients because it was unavailable during the study tenure. Despite these, the long-term results of endotherapy in PD are the least studied. To our knowledge, this is among the very few prospective studies with a good sample size identifying factors contributing to successful endotherapy.

## Conclusions

To summarize, symptomatic patients who have RAP with PD are most likely to have clinical benefits after endotherapy. The primary outcome of significant pain reduction was achieved in 69.1% of patients with an acceptable AE rate. Recurrent attacks of RAP after index ERCP and smoking negatively impact success of endotherapy. Early CP is modestly seen among RAP patients. If patients remain symptomatic or have recurrent attacks of pain after index endotherapy, they can be referred for surgery. Whether patients with infrequent episodes need aggressive therapy to prevent progression to CP needs to be studied in future prospective well-controlled trials.

### Data availability statement

The data underlying this article will be shared on reasonable request to the corresponding author.
